# Antenatal predictors of incident and persistent postnatal depressive symptoms in rural Ethiopia: a population-based prospective study

**DOI:** 10.1186/s12978-019-0690-0

**Published:** 2019-03-04

**Authors:** Tesera Bitew, Charlotte Hanlon, Girmay Medhin, Abebaw Fekadu

**Affiliations:** 1 Department of Psychology, Debre Markos University, Institute of Educational and Behavioural Sciences, Debre Markos, Ethiopia; 2 Department of Psychiatry, Addis Ababa University, College of Health Sciences, School of Medicine, Addis Ababa, Ethiopia; 3 King’s College London, Institute of Psychiatry, Psychology and Neuroscience, Centre for Global Mental Health, London, UK; 4 Centre for Innovative Drug Development and Therapeutic Trials for Africa (CDT-Africa), Addis Ababa University, Addis Ababa, Ethiopia; 5 Global Health & Infection Department, Brighton and Sussex Medical School, Brighton, UK; 6 Department of Psychological Medicine, King’s College London, Institute of Psychiatry, Psychology and Neuroscience, Centre for Affective Disorders, London, UK; 7 Aklilu Lemma Institute of Pathobiology, Addis Ababa University, Addis Ababa, Ethiopia

**Keywords:** Incidence, Persistent depression, Perinatal depression, Postnatal depression, Depressive symptoms, Ethiopia, Low and middle income countries

## Abstract

**Background:**

There have been few studies to examine antenatal predictors of incident postnatal depression, particularly in low- and middle-income countries (LMICs). The aim of this study was to investigate antenatal predictors of incident and persistent maternal depression in a rural Ethiopian community in order to inform development of antenatal interventions.

**Method:**

A population-based prospective study was conducted in Sodo district, south central Ethiopia. A locally validated version of the Patient Health Questionnaire (PHQ-9) was used to assess antenatal (second and third trimesters) and postnatal (4–12 weeks after childbirth) depressive symptoms, with a PHQ-9 cut-off of five or more indicating high depressive symptoms. Poisson regression with robust standard errors was used to identify independent predictors of persistence and incidence of postnatal depressive symptoms from a range of antenatal, clinical and psychosocial risk factors.

**Result:**

Out of 1311 women recruited antenatally, 1240 (356 with and 884 without antenatal depressive symptoms) were followed up in the postnatal period. Among 356 women with antenatal depressive symptoms, the elevated symptoms persisted into postnatal period in 138 women (38.8%). Out of 884 women without antenatal depressive symptoms, 136 (15.4%) experienced incident elevated depressive symptoms postnatally. The prevalence of high postnatal depressive symptoms in the follow-up sample was 274 (22.1%). Higher intimate partner violence scores in pregnancy were significantly associated with greater risk of incident depressive symptoms [adjusted Risk Ratio (aRR) = 1.06, 95% CI: 1.00, 1.12]. Each 1-point increment in baseline PHQ-9 score predicted an increased risk of incidence of postnatal depressive symptoms (aRR = 1.29, 95% CI: 1.15, 1.45). There was no association between self-reported pregnancy complications, medical conditions or experience of threatening life events with either incidence or persistence of depressive symptoms.

**Conclusion:**

Psychological and social interventions to address intimate partner violence during pregnancy may be the most important priorities, able to address both incident and persistent depression.

**Electronic supplementary material:**

The online version of this article (10.1186/s12978-019-0690-0) contains supplementary material, which is available to authorized users.

## Plain English summary

Postnatal depression is an important problem worldwide, affecting up to 1 in 5 women, especially in developing countries. It would be helpful to identify pregnant women who are at risk of postnatal depression so that they can be offered treatment and support, but there have been few studies from developing countries.

In this study, we followed up 1240 pregnant women from a rural community in Ethiopia to try to understand who would develop new symptoms of depression after child birth (2 to 3 months) or who would have depression symptoms that continued from pregnancy into the postnatal period. We measured depression using a questionnaire that had been shown to work in Ethiopia.

We found out that more than one-third of women had depression symptoms in pregnancy that continued into the postnatal period. For about one in seven women, depression symptoms only started after they had given birth. Domestic violence predicted both newly developing postnatal depression as well as depression that continued from pregnancy. Women who had even low levels of depression symptoms in pregnancy had higher risk of postnatal depression.

Identifying and addressing domestic violence in pregnancy is essential and may improve women’s postnatal mental health.

## Background

Perinatal depression affect 10 to 20% of women [1–3], with higher estimates during pregnancy [4, 5], especially in low and middle-income countries (LMICs) [1, 3, 6]. Antenatal depression has been associated with low birth weight [7, 8] and delayed initiation of breast feeding [9]. Antenatal depression has been shown to affect reproductive health through reduced dietary intake during pregnancy [10–12], and increased medical costs [13, 14] through increased emergency healthcare provider visits [15, 16], and perinatal complications [17], including prolonged labour [9, 18] and preeclampsia [18–21]. Postnatal depression has been associated with infant under-nutrition [12, 22], poorer child health [17, 23] and less optimal child development [24, 25]. In terms of the mother, both antenatal and postnatal depression are associated with increased risk of disability [6, 26, 27] and maternal morbidity and mortality [28] due to suicide [5].

Depression during pregnancy has been found to be the strongest predictor of postnatal depression [29–31] in a number of prospective studies and reviews examining the trajectory of perinatal depression [31, 32]. Antenatal depression persisted into the postnatal period in nearly half of prospectively observed samples of women [33–35]. In the general population, such persistent depression was associated with even greater disability, morbidity and mortality [14, 36].

In prospective studies from high-income countries, adversities such as intimate partner violence (IPV) [37], childhood adversities, life events during pregnancy [31, 38] and unplanned pregnancy [39] were risk factors for persistent perinatal depressive symptoms. The presence of previous mental health problems [31, 38] and development of psychological conditions during pregnancy such as low self-esteem, perceived isolation [38] and high anxiety during pregnancy [31, 38] also predicted persistent perinatal depressive symptoms. Increased exposure to threatening life events [1, 30, 40], IPV [1, 30, 40–42], reduced social support and low socio-economic status [1, 30, 40–42] have been found to be associated cross-sectionally with both antenatal and postnatal depression. However, there have been very few studies from LMICs where investigators attempted to prospectively disaggregate the predictors of persistent and incident postnatal depression within the same study. In a previous study from rural Ethiopia, lack of adherence to endorsed socio-cultural practices was associated with incidence and persistence of depression into the postnatal period [4], but the role of other antenatal clinical and social risk factors was not reported.

The aim of this study was to disaggregate the antenatal predictors of persistent and incident postnatal depressive symptoms. We hypothesised that having a comorbid medical condition, higher exposure to IPV, experience of threatening life events and pregnancy complications would predict both persistent and incident postnatal depressive symptoms after controlling for socio-demographic and socio-economic factors.

## Methods

### Study design

A population-based cohort study was conducted, with women recruited in the second or third trimester of pregnancy and reassessed in the community for depressive symptoms 4–12 weeks after childbirth.

### Study setting

The study setting was Sodo district, Gurage Zone of the Southern Nations, Nationalities and Peoples’ Region (SNNPR) of Ethiopia. The district is located about 100 km south of Addis Ababa, the capital of Ethiopia, and has diverse topographic and climatic conditions. At the time of the study, over 160,000 people lived in Sodo district, predominantly in rural sub-districts (54 rural and 4 urban) [43]. The main source of income for about 85% the population is subsistence agriculture [44].

The district has eight primary health care facilities (health centres) each for about 25,000 population and one district hospital [45]. Under each of the primary healthcare facilities, there are five health posts (one in each sub-district). Health posts are basic health facilities staffed by a pair of health extension workers (HEWs) who are responsible for delivery of core public health programmes, such as reproductive health including family planning, community hygiene and health education [45]. At the time this study was being conducted, a new district level mental health care plan was being implemented as part of the Programme for Improving Mental health carE (PRIME) [46]. In PRIME, mental health care was integrated into primary health care and maternal care services through training of primary health care workers and midwives.

### Participants

Participants were recruited into the cohort if the following eligibility criteria were fulfilled: being in the second or third trimester of pregnancy, having continuous residence in the area for at least the last six months); not having obvious hearing or intellectual impairment to the extent of hindering adequate communication and aged 15 years or more.

### Sample size

The sample size was estimated for another study which aimed to examine the association between antenatal depressive symptoms and maternal healthcare use [15] using EpiInfo version 7 [47] assuming statistical power of 80% and a 95% confidence interval. The estimated sample size was 1355 women who were identified within a three month period, between early September and end of December, 2014. Of these, 44 identified antenatal women were non-eligible [15] leaving a total of 1311 women who were invited to participate. All eligible participants were prospectively followed up until 4–12 weeks (a median of eight weeks) after delivery. Four weeks was considered to be the optimal time point to distinguish postpartum depression from postpartum blues as defined in the Diagnostic and Statistical Manual of the American Psychiatric Association (DSM IV) [48] and to reduce the chance of recall bias.

### Data collection procedures

The data collection procedures have been described in detail previously [15] and will be briefly summarised here. During participant recruitment, HEWs, health development army leaders (health education volunteers), sub-district chairmen and pregnant women themselves acted as key informants to identify pregnant women in the community [15]. Trained lay data collectors then made home visits to the identified women and informed them about the study, invited them to provide informed consent and conducted the interview in Amharic, the official language of the district.

### Data quality control

Data collection was conducted by 40 data collectors who had experience of mental health data collection in the same study setting and had a minimum educational level of grade 10. There were four supervisors (two diploma level and two with bachelor degree). Two days of training were given. The training focused on ensuring understanding of the questionnaire and the procedures of administering the questionnaire, with the aim of reducing social desirability and interviewer biases and to avoid potential ethical violations. Lectures, demonstration and role plays were used during the training. The coordinator of the study (TB) closely monitored the data collection process along with field supervisors. Completed questionnaires were carefully checked for consistency and missing data by the field supervisors, then by the coordinator of the study (TB) and finally by the data entry clerks. Questionnaires with inconsistencies or missing data were returned to data collectors for reinvestigation and correction. The same was done for the errors identified by data entry clerks provided that the items were not time sensitive. Data were double-entered using EpiData version 3.1 [47]. The STROBE guideline [49] was used for reporting.

### Assessments

Assessment of socio-economic and demographic characteristics, obstetric history and depressive symptoms was carried out during the second or third trimester of pregnancy (September – November 2014). Depressive symptoms were reassessed 4–12 weeks after childbirth.

### Outcome

Onset of new depressive symptoms (incident depression) and persistence of depressive symptoms (persistent depression) were the outcomes of interest. Incident depression was identified when women who had a Patient Health Questionnaire-9 (PHQ-9) score below five points (the optimal cut-off point for detecting major depressive disorder in a local validation) [50–52] antenatally and above the cut-off (five points or more) at the postnatal assessment. Persistent depression was identified when participants scored above the cut-off at both the antenatal and postnatal assessments. PHQ-9 is a fully structured questionnaire comprising nine questions about depressive symptoms based on the diagnostic criteria for major depressive disorder specified in the DSM IV [53].

### Predictors

Socio-economic and socio-demographic variables, including residence (rural vs. urban), marital status, family income and level of education were assessed at baseline. Family income was assessed by asking the participants about annual income, daily income or monthly income depending on their occupation (farming, trade, daily labour or employment) which was then converted into monthly income for all participants. IPV was assessed using a five item violence screening test, which was preferred due to the acceptability of the wording among antenatal women [54]. The 12 item list of threatening experiences (LTE) [55], a tool that has been employed in previous studies from Ethiopia, was used to assess the occurrence of stressful life events during the six months prior to assessment. Pregnancy intention was asked using an item taken from the Ethiopian Demographic Health Survey [43]. The item asks whether a woman wanted the pregnancy (‘wanted’) or wanted to have delayed it (‘mistimed’) or didn’t want to have it at all (‘unwanted’).

The number of chronic medical conditions such as tuberculosis, HIV/AIDS, renal disease and cardiac diseases that were diagnosed by primary healthcare providers and communicated to patients was also assessed at baseline through self-report. The number of previous adverse perinatal outcomes, including spontaneous abortion, stillbirth and neonatal mortality was obtained through self-report. Finally, pregnancy complications were measured by asking about the woman’s experience of key danger signs of obstetric complications in the current pregnancy: bleeding, swollen hands/face, blurred vision, convulsions, high fever, loss of consciousness, severe abdominal pain, waters breaking 12 h before childbirth, discharge with unusual odour, pain during urination, severe headache and severe weakness which were recorded as “none” versus “one or more”. Women were informed about these danger signs by the health worker when they attend for antenatal care [17].

### Data analysis

Stata version 13 was used to analyse the data. The educational status of the participants was categorized as “non-literate”, “primary schooling” and “secondary schooling”, while marital status was grouped into two categories: married and single (unmarried, widowed and divorced). Monthly income was categorized into tertiles as “low”, “medium” and “high” income categories.

Women with persistent depressive symptoms were coded as “1” while those with non-persistent antenatal depressive symptoms (symptoms during pregnancy alone) were coded as “0”. Out of those women without any depressive symptoms during pregnancy, those with incidence after childbirth were coded as “1” while those without any new symptoms were coded as “0”. A Poisson regression working model with robust standard error was employed to examine antenatal risk factors for persistent and incident depressive symptoms. Adjustment for the follow up period was made in all regression models and a collinearity test for independent variables was performed. The Poisson regression was preferred to logistic regression as logistic regression can inflate the true effect size in situations when there is a high prevalence of the outcome [56]. Poisson regression gives an estimate of the risk ratio [56]. Complete case analysis was used as the amount of missing data (5.4%) was very small and unlikely to impact upon the results [57, 58].

## Results

### Characteristics of the respondents

**Fig. 1:**
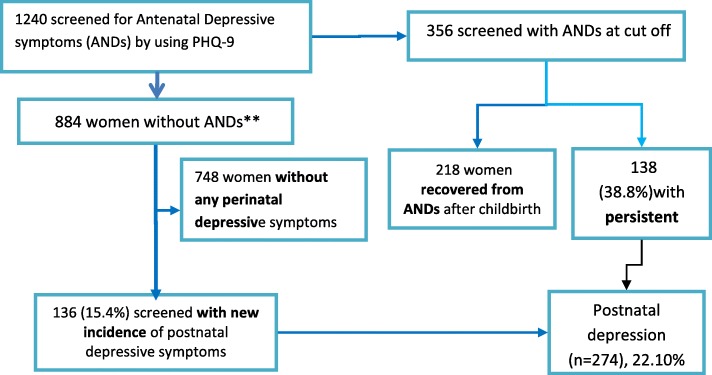
Diagram to show recruitment procedure of study participants

From a total of 1311 eligible women recruited during pregnancy [15], 1240 (94.6%) women were available for the follow-up assessment. Reasons for non-availability were: refusal (*n* = 11), not being traceable after birth (*n* = 27); had not delivered by the end of the scheduled follow-up (*n* = 30); death during labour (*n* = 1) and missing and un-interpretable data (n = 2). The remaining 1240 women were assessed at an average of eight weeks after birth (25th centile = 6 weeks and 75th centile = 11 weeks) (Fig. 1). The mean age of participants was 26.8 years and 19.4% were primigravida. Most women were married (98.8%), non-literate (67.8%) and residing in a rural area (92.1%) (Table 1). There was no significant difference between women who were followed up and those lost to follow-up with respect to most key baseline characteristics (Additional file 1: Table S1).

**Table 1:** Baseline participant characteristics

		Baseline (*N* = 1311)		Followed up (*N* = 1240)	
Variables	Values	PHQ-9 < 5 N (%)	PHQ-9 ≥ 5 N (%)	PHQ-9 < 5 N (%)	PHQ-9 ≥ 5 N (%)
Pregnancy Intention	Wanted	552 (59.7)	182 (47.0)	528 (59.7)	168 (47.2)
	Mistimed	66 (7.1)	36 (9.3)	61 (6.9)	30 (8.4)
	Unwanted	306 (33.1)	169 (44.7)	295 (33.4)	158 (44.4)
Monthly Income category	Low	286 (31.0)	170 (43.9)	276 (31.2)	159 (44.4)
	Medium	300 (32.5)	123 (31.8)	283 (32.0)	109 (30.6)
	High	335 (36.3)	94 (24.3)	325 (36.8)	89 (25.0)
Residence	Urban	87 (9.4)	16 (4.1)	82 (9.3)	16 (4.5)
	Rural	837 (90.6)	371 (95.6)	802 (90.7)	340 (95.5)
Mother’s Education	Non-literate	600 (64.9)	278 (71.8)	581 (65.7)	260 (73.0)
	Literate	334 (36.1)	109 (28.2)	303 (34.3)	96 (27.0)
Number of Chronic Illnesses	None	578 (62.6)	207 (53.5)	554 (62.7)	188 (52.8)
	One or more	346 (37.4)	180 (46.5)	330 (37.3)	168 (47.2)
History of adverse perinatal outcomes	None	723 (76.1)	252 (65.1)	674 (76.2)	233 (65.4)
	One or more	221 (23.9)	135 (34.9)	210 (23.8)	123 (34.6)
Self-reported pregnancy complications	None	567 (61.4)	89 (23.0)	541 (61.2)	86 (24.2)
	One or more	357 (38.6)	298 (77.0)	343 (38.8)	270 (75.8)
Alcohol Use	Nil (none use)	614 (66.5)	261 (67.4)	588 (66.5)	240 (67.4)
	Minimal (1–2 points	264 (28.6)	95 (24.5)	251 (28.4)	87 (24.4)
	Hazardous (3 or more points)	46 (5.0)	31 (8.0)	45 (5.1)	29 (8.1)

Income was categorized into tertiles as low, medium and high

Intimate Partner violence score: minimum = 9; Maximum = 16; Mean = 2.14; SD = 2.88

Parity score: minimum = 0; Maximum = 12; Mean = 2.74; SD = 2.09

Among those women assessed at both time points (*n* = 1240), a total of 356 women (28.7%) had high antenatal depressive symptoms (≥5 on the PHQ-9) and the remaining 884 women had low antenatal depressive symptoms (≤4 on PHQ-9). Of those women with high antenatal depressive symptoms, 138 (38.8%) persisted into the postnatal period. In women with low antenatal depressive symptoms, 136 (15.4%) developed newly elevated depressive symptoms (incident cases) (PHQ-9 ≥ 5) during the postnatal period. The prevalence of postnatal depressive symptoms was 22.1% (combining both incident and persistent cases) (Fig. 1).

### Predictors of persistence and incidence of maternal depressive symptoms

Table 2 shows the predictors of persistence and incidence of maternal depressive symptoms. A one point increment in intimate partner violence (IPV) score marginally predicted persistence [adjusted Relative Risk (aRR) 1.03, 95% CI: 0.99, 1.07) of maternal depressive symptoms compared to women whose depressive symptoms was resolved after their childbirth. IPV also predicted greater risk of incidence (aRR = 1.06, 95% CI: 1.00, 1.12) of postnatal depressive symptoms. Each increment in sub-threshold antenatal PHQ-9 score predicted increased risk of incidence of postnatal depressive symptoms (aRR = 1.29, 95% CI: 1.15, 1.45).

**Table 2:** Predictors of persistence and incidence of maternal depressive symptoms

	aRR (95% CI)	
Predictors	Persistent vs non-persistent, *N* = 356	Incidence vs persistently negative, *N* = 884
Pregnancy Intention: Wanted	1	1
Mistimed	0.77 (0.40, 1.47)	0.75 (0.36, 1.56)
Unwanted	1.11 (0.81, 1.51)	1.12 (0.79, 1.58)
Family Income: Low	1	1
Medium	1.14 (0.83, 1.57)	1.31 (0.91, 1.90)
High	1.12 (0.78, 1.61)	0.85 (0.55, 1.31)
Residence: Rural	1.34 (0.59, 3.06)	0.69 (0.40, 1.21)
Mother’s Education: Non-literate	1	1
Literate	0.96 (0.67, 1.37)	0.91 (0.60, 1.37)
PHQ-9 total score	1.03 (0.99, 1.06)	1.29 (1.15, 1.45)*
Number of chronic conditions	1.17 (0.97, 1.41)	1.13 (0.86,1.48)
History of adverse perinatal outcome	0.89 (0.66, 1.20)	1.09 (0.76, 1.56)
Self-reported pregnancy complications	1.04 (0.73, 1.47)	1.24 (0.90, 1.71)
Intimate Partner Violence Score	1.03 (0.99, 1.07)	1.06 (1.00, 1.12)*
Number of threatening life events	0.97 (0.89, 1.06)	1.03 (0.92, 1.14)
Parity	1.05 (0.98, 1.13)	0.97 (0.88, 1.07)
Alcohol use: None	1	1
Minimal	0.89 (0.63, 1.26)	0.88 (0.61, 1.25)
Hazardous	1.28 (0.82, 1.98)	1.11 (0.55, 2.23)

* = significant at < 0.05

Having one or more symptoms of pregnancy complications predicted incidence of postnatal depressive symptoms in the non-adjusted model. But, the association disappeared (aRR = 1.24, 95% CI: 0.90, 1.71) when total antenatal depressive symptoms score was included in the model. Unwanted pregnancy (aRR = 1.11, 95% CI: 0.81, 1.51), having medical conditions (aRR =1.17, 95% CI: 0.97, 1.41) and number of threatening life experiences (aRR = 0.97, 95% CI: 0.89, 1.06) were not significantly associated with persistent depressive symptoms.

## Discussion

This is one of the very few studies to have investigated factors associated with persistent and incident depression during the perinatal period in a LMIC setting. The key findings are: [1] nearly two-fifths of women with antenatal depressive symptoms had persistent depressive symptomatology; [2] about a sixth (15.4%) of those without depression antenatally had new onset of depressive symptoms postnatally; [3] the potential risk factors for incident and persistent perinatal depression are IPV and sub-threshold antenatal PHQ-9 symptom score. Our finding of a high prevalence of persistent depressive symptoms from pregnancy to the postnatal period is in keeping with findings from studies conducted in high income countries [31, 38, 39]. However, our finding differs from a previous study from rural Ethiopia where the persistence of antenatal depressive symptoms was lower than expected (21.4% of women with high antenatal depression had high scores postnatally) [4]. The difference may be due to the instruments used to assess depressive symptoms. However, in both Ethiopian studies, the extent of persistence was much lower than has been seen in some studies from south Asia [59]. The increased risk of incidence and persistence of maternal depressive symptoms in women exposed to intimate partner violence (even after controlling for adverse perinatal outcomes) supports previous findings of an association between psychosocial factors [31], like IPV, with antenatal depression [60–64] and persistence of depression in longitudinal studies after childbirth [31, 38]. The high prevalence of IPV and its association with antenatal depression has also been well established in several LMIC settings [1, 41, 42]. However, in the current study we were able to demonstrate that IPV was a risk factor for incident postnatal depressive symptoms. Reduced communication and resulting non-endorsement of socio-cultural practices, activities that predict having postnatal common mental disorders [4], may explain the increased risk of incident and persistent depressive symptoms among women with increased IPV. Secondly, IPV could potentially affect persistence of antenatal depressive symptoms to postnatal period through circular causality, where IPV potentially lowers women’s mood and so worsens their communication abilities with their partners and ultimately maintains persistence of depressive symptoms and also bringing new incident cases into the loop.

Thirdly, IPV and other situational challenges such as stressful life events could potentially predict components of perinatal depressive symptoms through biological pathways in which IPV might activate the Hypothalamico- Pituitary Adrenal Axis (HPA) to produce emotion arousing hormones that could have a link with depressive symptoms [65, 66].

Increased risk of incidence of postnatal depressive symptoms associated with sub-threshold antenatal PHQ-9 scores in the current study is an important finding, which underscores the need for further clinical consideration of prevention or early intervention for women with sub-threshold PHQ-9 scores. This finding supports increased risk of incidence of depressive symptoms among women with anxiety disorders prior to pregnancy [38]. The risk of persistent perinatal depression associated with poverty [8, 67] and unwanted pregnancy [39] in previous studies was not replicated in the current study. This may be because IPV could be- a gateway through which these socioeconomic and empowerment-related factors affect depression and this was controlled for statistically in our study.

One of the strengths of our study is that it is a population-based prospective study with low chance of selection and reverse causality biases. We also used a locally validated measure of perinatal depressive symptoms and distinguished persistent depressive symptoms from incident postnatal symptoms in order to distinguish predictors of new onset from predictors of persistence while adjusting for follow up period and socio-demographic characteristics. However, we could not distinguish relapses of antenatal depressive symptoms in the postnatal period from persistence of antenatal depressive symptoms into postnatal period. We rather defined any woman screened with depressive symptoms both at antenatal and postnatal periods as having ‘persistent depressive symptoms’. Rates of persistence and incidence have to be understood with caution considering the low positive predictive value of the PHQ-9 [68]. Also, we were not able to determine whether women who were referred to PHC for their severe levels of antenatal depression had received treatment (counselling, antidepressant), which could potentially have reduced the rate of persistence.

## Conclusion

Antenatal depressive symptoms persisting into the postnatal period accounted for nearly two-fifths of the prevalence of postnatal depressive symptoms implying the need to prioritize interventions strategies for antenatal depressive symptoms to prevent persistence. The findings support a problem-solving approach to sub-threshold depressive symptoms and psychosocial stressors in the antenatal period, especially intimate partner violence, along with comprehensive care for pregnancy complications. Studies that focus on the association between the HPA (Hypothalamus-Pituitary Axis) and depression may also consider intimate partner violence as a potential confounder of the association between HPA and depression.

## Additional file

Additional file 1: **Table S1.** Characteristics of followed up and lost to follow up participants in respect to baseline measures. (DOCX 17 kb)

## Abbreviations

aOR: Adjusted Odds Ratio
aRR: Adjusted Risk Ratio
cOR: Crude Odds Ratio
cRR: Crude Risk Ratio
HEWs: Health Extension Workers
HICs: High Income Countries
IPV: Intimate Partner Violence
LMICs: Lower Middle Income Countries
LTE: List of Threatening Events
PHQ-9: Patient Health Questionnaire-9
RR: Risk Ratio
T.B.: Tuberculosis
