# Supplementary material for: Antenatal predictors of incident and persistent postnatal depressive symptoms in rural Ethiopia: a population-based prospective study
Source: Reprod Health. 2019 Mar 4;16:28. doi: 10.1186/s12978-019-0690-0 (PMC6399829; doi:10.1186/s12978-019-0690-0)
Supplement: Supplementary file 1 — Table S1. Characteristics of followed up and lost to follow up participants in respect to baseline measures. (DOCX 17 kb) [file 12978_2019_690_MOESM1_ESM.docx]

Additional file 1: Table S1

Characteristics of followed up and lost to follow up participants in respect to baseline measures

|  | | **Follow up** | **Loss to follow up** | **Pearson X^2^/ t (p-value)** |
| --- | --- | --- | --- | --- |
| **Variables Values** | | **N (%)** | **N (%)** |  |
| Pregnancy Intention | Wanted | 696 (56.1) | 38 (53.5) | x^2^= 6.39 ( 0.041) |
|  | Mistimed | 91 (7.3) | 11 (15.5) |  |
|  | Unwanted | 453 (36.5) | 22 (31.0) |  |
| Monthly Income category | Low | 434 (35.0) | 25 (35.2) | x^2^= 6.11 (0.047) |
|  | Medium | 392 (31.6) | 31 (43.7) |  |
|  | High | 414 (33.4) | 15 (21.1) |  |
| Residence | Urban | 98 (7.9) | 5 (7.0) | x^2^= 0.07 (0.793) |
|  | Rural | 1142 (92.1) | 66 (93.0) |  |
| Mother's Education | Non-literate | 841 (67.8) | 37 (52.1) | x^2^= 7.49 (0.006) |
|  | Literate | 399 (32.2) | 34 (47.9) |  |
| Number of Chronic Illnesses | None | 742 (59.8) | 43 (60.5) | x^2^= 0.02 (0.904) |
|  | One or more | 498 (40.2) | 28 (39.5) |  |
| History of perinatal complications | None | 907 (73.2) | 48 (67.6) | x^2^= 1.042 (0.307) |
|  | One or more | 333 (26.8) | 23 (32.4) |  |
| Self-reported pregnancy complications | None | 627 (50.6) | 29 (40.8) | x^2^= 2.54 (0.111) |
|  | One or more | 613 (49.4) | 42 (59.1) |  |
| Alcohol Use | Nil (none use) | 828 (66.8) | 47 (66.2) | x^2^= 0.48 (0.786) |
|  | Minimal (1-2 units) | 338 (27.3) | 21 (29.6) |  |
|  | Hazardous (3 or more units) | 74 (6.0) | 3 (4.2) |  |
| PHQ-9 score |  | Mean = 3.60 | Mean =4.37 | t = -1.58 (0.113) |
| Intimate partner violence score |  | Mean = 2.14 | Mean = 2.61 | t= -1.32 (0.186) |
| Parity |  | Mean =2.74 | Mean = 1.90 | t = 3.28 (0.001) |
